# Registration of 3D fetal neurosonography and MRI^☆^

**DOI:** 10.1016/j.media.2013.07.004

**Published:** 2013-12

**Authors:** Maria Kuklisova-Murgasova, Amalia Cifor, Raffaele Napolitano, Aris Papageorghiou, Gerardine Quaghebeur, Mary A. Rutherford, Joseph V. Hajnal, J. Alison Noble, Julia A. Schnabel

**Affiliations:** a Institute of Biomedical Engineering, Department of Engineering Science, University of Oxford, UK; b Nuffield Department of Obstetrics and Gynecology, John Radcliffe Hospital, Oxford University Hospitals NHS Trust, Oxford, UK; c Neuroradiology, John Radcliffe Hospital, Oxford University Hospitals NHS Trust, Oxford, UK; d Department of Perinatal Imaging and Health, King’s College London, UK; e Department of Biomedical Engineering, King’s College London, UK; f Centre for the Developing Brain, King’s College London, UK

**Keywords:** Fetal 3D ultrasound, MR ultrasound registration, Fetal neurosonography, Block matching

## Abstract

We propose a method for registration of 3D fetal brain ultrasound with a reconstructed magnetic resonance fetal brain volume. This method, for the first time, allows the alignment of models of the fetal brain built from magnetic resonance images with 3D fetal brain ultrasound, opening possibilities to develop new, prior information based image analysis methods for 3D fetal neurosonography. The reconstructed magnetic resonance volume is first segmented using a probabilistic atlas and a pseudo ultrasound image volume is simulated from the segmentation. This pseudo ultrasound image is then affinely aligned with clinical ultrasound fetal brain volumes using a robust block-matching approach that can deal with intensity artefacts and missing features in the ultrasound images. A qualitative and quantitative evaluation demonstrates good performance of the method for our application, in comparison with other tested approaches. The intensity average of 27 ultrasound images co-aligned with the pseudo ultrasound template shows good correlation with anatomy of the fetal brain as seen in the reconstructed magnetic resonance image.

## Graphical abstract

**Figure:**
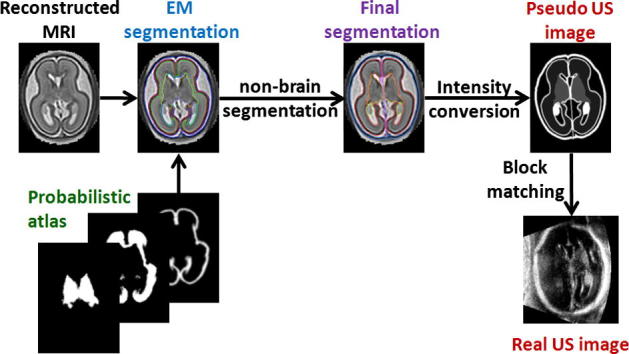

**Highlights:**

• A novel method for affine registration of fetal neurosonography and brain MRI proposed.
• Conversion of fetal MRI into pseudo US image described.
• All data were successfully aligned using robust block-matching approach.
• Average of 27 US volumes revealed near-complete anatomy of the fetal brain.

## 1 Introduction

### 1.1 Motivation

Fetal ultrasound (US) is the imaging modality of choice in clinical practice for assessing fetal development. Traditional methods for assessment of fetal brain development rely on qualitative evaluation and manual measurements performed on 2D US scans, where a pre-defined plane is manually selected by the sonographer, and several 2D measurements are taken to assess the size of the fetal head and some brain structures (ISUOG, 2007). If a brain abnormality is suspected, fetal magnetic resonance (MR) imaging is often performed to confirm the finding. US does not always depict sufficient information about the structures within the fetal brain, largely due to acoustic shadows caused by the fetal skull, while MR imaging is unaffected by the presence of bone (Pugash et al., 2008). Recent work, however, provides evidence that in prospective studies fetal brain structures and anomalies can be visualised correctly in 90% of the cases by experienced operators with 3D US (Gandolfi Colleoni et al., 2012). 3D fetal neurosonography is currently one of the most active research fields in obstetric imaging.

Recently, large databases of longitudinal 3D neurosonography scans are becoming available thanks to initiatives Intergrowth-21st^1^ and Interbio-21st.^2^ The Intergrowth-21st consortium collected several thousands of normal fetal US scans, including 3D brain US, containing several thousands of subjects scanned at up to six time-points during pregnancy, from eight different sites around the world. This database is to serve for development of new “prescriptive” standards describing normal fetal growth. The aim of Interbio-21st study is to collect similarly large number of fetal scans, to improve the phenotypic characterisation of the intrauterine growth restriction/small for gestational age and preterm birth syndromes, so as to develop better strategies to correct the short and long-term effects of an adverse intrauterine environment. To fully exploit this wealth of information, development of tools for image analysis of fetal 3D US becomes of very high importance.

In this paper we propose a method for the alignment of fetal brain 3D US and MR images, which will in future allow us to explore the idea, that models of brain anatomy build from more complete MR images of fetal brain can be exploited to serve as prior knowledge for automatic image analysis of fetal brain 3D US or to assist in making clinical diagnosis from 3D fetal neurosonography. Additionally, intra-subject alignment of fetal 3D US and MR images can facilitate clinical studies to determine whether fusion of information from fetal brain MR and US images could enhance abnormality screening. It can also facilitate use of MR imaging for validation of quantitative measurements performed using 3D US.

### 1.2 Related work

To our knowledge there is no prior literature on registration of fetal brain MR and US images, except for our recent works (Kuklisova-Murgasova et al., 2012b,a) on which this paper builds. The methods proposed in literature mostly focus on registration of pre-operative MRI/CT and real-time US, usually aimed at adult organs, such as brain, liver or heart (Roche et al., 2001; Penney et al., 2004; Arbel et al., 2004; Blackall et al., 2005; Mellor and Brady, 2005; Zhang et al., 2007; Brooks et al., 2008; Wein et al., 2008; Hu et al., 2009; Milko et al., 2009; King et al., 2009). The major challenge in aligning MR (or CT) and US images is that there does not exist a simple intensity mapping between the two modalities. Authors therefore employ various strategies to extract corresponding features from both modalities such as gradient magnitude (Roche et al., 2001; Brooks et al., 2008), sheet-like features (Hu et al., 2009), local phase (Mellor and Brady, 2005; Zhang et al., 2007) or segmentation of different structures (Arbel et al., 2001; Penney et al., 2004; Arbel et al., 2004; Blackall et al., 2005; Milko et al., 2009; King et al., 2009). Wein et al. (2008) performs simulation of US from CT of liver and registers simulated and real US images.

Registration of brain MR and US images was previously proposed for image guided neurosurgery in adults to non-rigidly correct for brain-shift during surgery (Arbel et al., 2001, 2004) as well as for rigid alignment of adult or neonatal brains (Roche et al., 2001; Ji et al., 2008; Mercier et al., 2012). Roche et al. (2001) suggested to estimate a non-linear relationship between MR image intensities and gradient magnitude, and US image intensities, using generalised correlation ratio (CR) as a similarity measure. Arbel et al. (2001, 2004) proposed to perform segmentation of the brain MR image (MRI) followed by simulation of a pseudo US image which is then non-rigidly registered with the US image using local normalised cross-correlation (NCC) as a similarity measure. The rigid alignment is performed by first aligning the MR image into the patient space by manually selecting the landmark points in the MR image and on the patient. The US images are then aligned with the MRI using information from a tracker device. Arbel’s work was later extended by Mercier et al. (2012), who proposed to improve the initial rigid alignment of pre-operative MRI with US using automatic registration of pseudo US image with the real US image. The authors show that this method improved the initial rigid alignment and also outperformed mutual-information-based automatic alignment of original MRI and US, proposed by Ji et al. (2008).

US images contain intensity artefacts, such as acoustic shadows, attenuation, and reverberations, which may negatively affect performance of the registration methods. During cranial sonography in adults and neonates, a sonographer can position the probe next to the opening in the skull and thus avoid most artefacts which cannot be avoided in fetal neurosonography. In fetal neurosonography, on the other hand, the most pronounced artefacts appear due to difficulties in positioning the probe and interference with maternal tissues. These include shadows caused by presence of fetal skull and reverberations – high signal corrupting the fetal brain image, caused by multiple reflections of the US beam by fetal skull and maternal tissues, resulting in missing features and variable signal strength. Wein et al. (2008) suggested to estimate the attenuation and shadows from knowledge of physical properties of the scanned organ. The method was developed for registration CT and US images of liver and thus could take advantage of excellent visibility of bone in CT, creating ideal situation for estimation of shadows. Additionally, the signal strength in CT can be directly related to the acoustic impedance of the tissues. This property was also exploited in their work for estimation of reflections of the US beam. The echogeneity of different tissues, or speckle, however, cannot be easily simulated from CT. Wein et al. (2008) therefore uses correlation ratio to estimate functional relationship between regional echogeneity in US and CT signal, a similarity measure previously proposed by Roche et al. (2001) for registration of adult and neonatal brain MRI and US during image-guided neurosurgery.

MRI, however, does not possess such favourable properties of CT for simulation of US images and estimation of the artefacts. Bone does not produce any MR signal and is therefore indistinguishable from the air or other tissues which appear dark. Additionally, the only partly calcified and not completely fused fetal skull causes rather complex pattern of shadows and signal loss. This, together with no currently existing models of developing fetal skull which could be used as a prior knowledge, renders automatic estimation of shadows (or reverberations) unfeasible at present. Unlike the previously proposed methods, we will have to rely on registration methodology robust towards missing features and intensity artefacts in US images. We propose to employ robust block-matching algorithm (Ourselin et al., 2001).

Another major challenge when aligning fetal brain MR and US images is the choice of a suitable similarity measure. The relationship between intensities in the fetal brain MR and US images is difficult to express. Features of the fetal brain visible in these modalities in relation to the anatomy have been well described in the clinical literature (Monteagudo and Timor-Tritsch, 2009; Rutherford, 2009; Glenn, 2010). While MRI offers good contrast between soft tissues, especially white matter (WM), grey matter (GM) and cerebro-spinal fluid (CSF), the WM-GM boundary does not appear in US at all. Additionally, the anatomical structures that dominate fetal US images, such as the choroid plexus, skull and falx are relatively poorly defined in fetal MRI (see Section 2.1). One strategy is to use simple multimodal similarity measure such as normalised mutual information (e.g. Ji et al. (2008)). In this case the similarity measure will match regional echogeneity of the US image with the MR intensities of the tissues and high intensity signals at the boundaries of the structures, characteristic of US images will be ignored. Mercier et al. (2012) showed that such approach can be rather unstable, which is consistent with the results presented in our previous work (Kuklisova-Murgasova et al., 2012b). Roche et al. (2001) proposed to include gradient of the MR image in a generalised CR. Though gradient image would contain some important features, such as brain surface, it would also include WM/GM boundary which is not visible in fetal US.

Alternative multimodal approach is to use robust block-matching algorithm with CR as a multimodal similarity measure (Ourselin et al., 2001). Our previous experiments (Kuklisova-Murgasova et al., 2012a) demonstrated good performance of this method, though we also showed that more tailored similarity measure can further improve the results.

Alternative to direct multimodal registration is simulation of the US from MRI followed by mono-modal registration. As we already argued, realistic simulation of fetal brain US from MRI is currently unfeasible, but the main features of fetal brain US can be created from segmented MRI and converted to a pseudo US image, as proposed by Arbel et al. (2001, 2004) and Mercier et al. (2012) for adult brain in context of computer assisted neurosurgery. Though this approach requires segmentation of the brain structures in MRI, which results in more complex pipeline of methods, pseudo US image offers better correspondences for matching with US than original MRI (Mercier et al., 2012; Kuklisova-Murgasova et al., 2012b,a). Building on the knowledge of how fetal anatomy typically appears in MRI and US (Monteagudo and Timor-Tritsch, 2009; Rutherford, 2009; Glenn, 2010), we are able to propose a similar pseudo US image construction for fetal brain MRI. By careful comparison of fetal brain MRI and US, we selected a set of structures important to be segmented for our task. This set is different from those chosen by Arbel or Mercier, as it includes some additional structures such as skull, falx, choroid plexus, brainstem and cerebellum. Segmentation of brain structures in fetuses and pre-term neonates has been previously described by several authors (Habas et al., 2010; Kuklisova-Murgasova et al., 2011a; Serag et al., 2012). To create the pseudo US image we need to extend this methodology to non-brain structures that form important landmarks in fetal brain US and help us to construct a more realistic pseudo US image. Our pseudo US image is independent of the position of the probe, unlike the one proposed in the work of Arbel, which makes the application of the method simpler in the cases like ours, where one of the goals is to process a large database of fetal US scans and recovering position of the probe might prove a rather difficult task.

The final consideration needs to be given to the registration algorithm. During image-guided neurosurgery, the rigid alignment of MR and US images is usually assisted using a tracking device. This information is not available in our database of fetal US. During fetal scanning, the sonographer attempts to acquire the 3D scan in consistent orientation with respect to the fetal brain. This can only be achieved with variable accuracy, depending on the position of the fetus. We observed that in our dataset used for evaluation, automatically recovered transformations contained rotations of up to 30° compared to the position of the template MRI. As we demonstrate in this paper, employing a block-matching algorithm (Ourselin et al., 2001), as opposed to registration using gradient descent optimisation, proves essential for a robust performance at this task.

### 1.3 The proposed method

In this paper we propose a method for the alignment of fetal brain US and MR volumes, designed to resolve the difficulties described in the previous section. We present a complete pipeline which successfully fulfils this task, by putting together building blocks of recently developed state-of-the-art methodology of fetal imaging, namely 3D US imaging of the fetal brain, structural MR imaging of the fetal brain, and reconstruction of fetal MRI volumes, with carefully chosen image analysis methodology for segmentation of fetal brain structures in MRI using fetal/pre-term brain atlases, and registration using a robust block-matching algorithm.

**Fig. 1:**
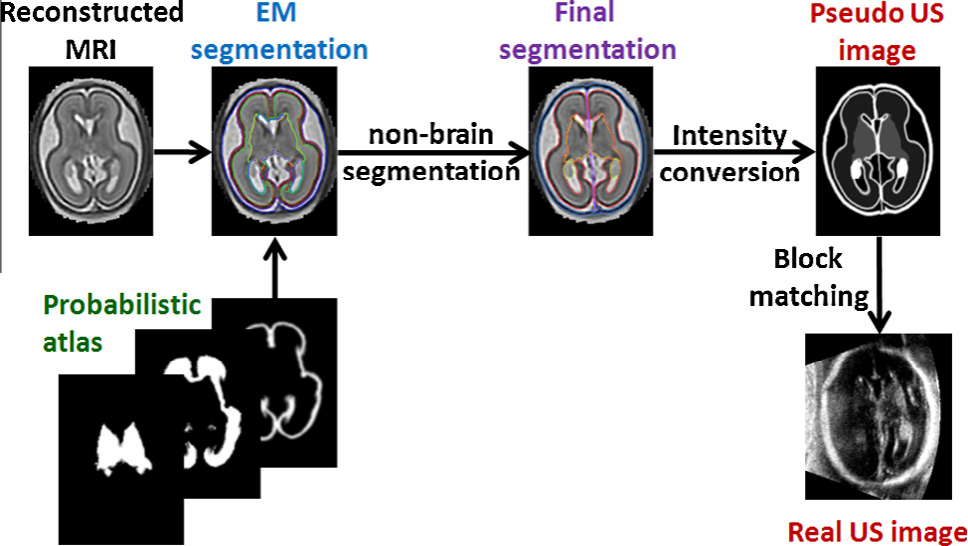
Overview of the proposed method.

High-resolution fetal brain MRI is first reconstructed from thin-slice acquisitions using our previously proposed method (Kuklisova-Murgasova et al., 2012c), see Section 2.2. The structures selected in Section 2.1 are then segmented in MRI. The brain structures are segmented using EM-based method and a probabilistic atlas (Kuklisova-Murgasova et al., 2011a), see Section 2.3, which is followed by segmentation of non-brain structures (Section 2.4). Though the segmentation of an MR template includes some manual steps, this needs to be done only once and the additional MR images can be segmented using fully automatic pipeline presented in Section 2.5. Segmentation of brain and non-brain structures is then converted into the pseudo US image, as described in Section 2.6. In this paper we use the term pseudo US image to refer to an image containing anatomical brain and head structures typically visible in fetal US but without speckle and artefacts such as shadows, attenuation and intensity variations due to angle of the ultrasound beam. This pseudo US image is then registered to the fetal neurosonography (Section 2.7), using robust block-matching algorithm (Ourselin et al., 2001). Our experiments presented in Sections 4 and 5 show good performance of the proposed method in inter-subject alignment of clinical 3D neurosonography images with fetal brain MRI. An overview of the method is presented in Fig. 1.

The methodology and results described in this paper were partly presented in our previous conference papers (Kuklisova-Murgasova et al., 2012b,a). In the first paper (Kuklisova-Murgasova et al., 2012b) we presented the idea to convert fetal MRI into pseudo US image. The pseudo US image was then registered with real US images using global NCC as a similarity measure and gradient descent as optimisation method. Our preliminary results on four US and one MR image of gestational age (GA) 28–29 weeks showed that performance was superior to multi-modal registration using NMI, conclusion similar to the one reached by Mercier et al. (2012). In the second paper (Kuklisova-Murgasova et al., 2012a) we described creation of pseudo US images at earlier GA (around 20 weeks) and improved robustness of the method by introducing robust block-matching algorithm (Ourselin et al., 2001), which employs local NCC as a similarity measure to deal with intensity and contrast variation and robust least trimmed squares to remove outliers produced by incorrectly matched blocks, which is helpful in situations when the corresponding features in US image is missing. We showed that this method performed better than multimodal block-matching with CR as a similarity measure. The segmentation pipeline proposed in this previous work contained some manual steps. In this paper we further develop this method (Kuklisova-Murgasova et al., 2012a) by presenting a fully automatic segmentation pipeline (Section 2.5) which has also been quantitatively evaluated in Section 5.3. The method, originally applied to images of approximately 20 weeks GA, has now also been applied to another time-point, approximately 29 weeks GA (Section 5.4). In Section 5.2 we also show, that the proposed method, registration using pseudo US image and block-matching algorithm with local NCC as similarity measure, outperforms pseudo US image-based methods with gradient descent optimisation and global as well as local NCC as a similarity measure, for our fetal application.

## 2 Methods

### 2.1 Features of fetal brain US and MRI

**Fig. 2:**
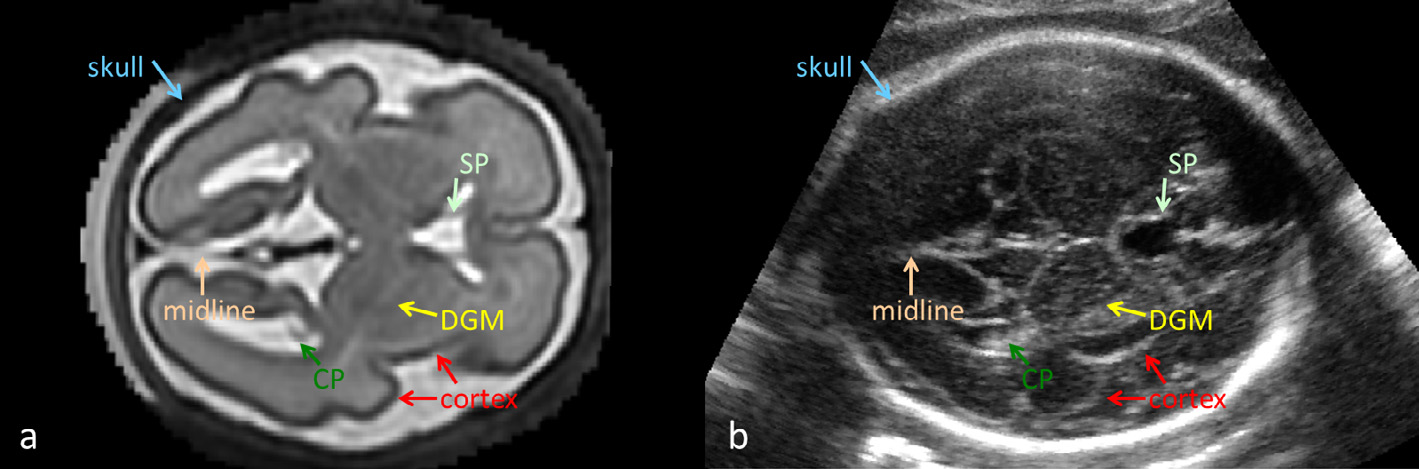
Features visible in fetal brain US in axial view: skull, cortical surface, midline (falx), DGM, choroid plexus (CP) and septum pellucidum (SP). Positions of these features in MRI (a) and in US (b). The MR and US images presented here are scans of different subjects with similar GA.

Alignment of fetal brain US and MRI requires a similarity measure describing the relationship between the structures visible in these two modalities. However, fetal brain MRI and US often depict complementary features. The MR signal is related to intrinsic tissue properties described by T1 and T2 relaxation times and usually offers good contrast between WM, GM and CSF. The US signal, on the other hand, is mainly created by the reflection of the acoustic beam due to the difference in acoustic impedance between different tissues, or the microstructure of the tissue. The features best detected using US are often hard to detect on MRI, because of its lower spatial resolution. Conversely, the WM-GM boundary does not appear in US at all. The features of the two modalities are compared in Fig. 2. To be able to correctly relate MR and US images, we create a pseudo US image from an MR image. For this purpose we choose the most pronounced features of a fetal brain depicted in 3D US (Monteagudo and Timor-Tritsch, 2009):

1. the skull,
2. the falx (midline membrane separating the two halves of the brain),
3. the brain surface,
4. the choroid plexus (located in the ventricles),
5. septi pellucidi (membranes separating ventricles),
6. cerebellum,
7. deep GM.

These anatomical structures are segmented in a fetal MRI and the segmentation is then converted into a pseudo US image. The features of the pseudo US image can then be correlated with the features of the real US image using a standard similarity measure, such as local NCC.

### 2.2 Reconstruction of fetal brain MRI

Fetal brain US often depicts structures that are difficult to visualise on MRI (e.g. falx), thus a high resolution reconstruction of a fetal brain MR image is essential for the creation of a pseudo US image. The MRI volume is therefore reconstructed from thin-slice data by iterating between a super-resolution reconstruction and a slice-to-volume rigid registration using our previously developed fetal MR reconstruction method (Kuklisova-Murgasova et al., 2011b, 2012c). The similarity measure used for slice-to-volume registration is normalised mutual information (Studholme et al., 1999). Our super-resolution reconstruction is designed to deal with the common artefacts of MR acquisition, namely exclusion of motion-corrupted slices, intensity matching and bias field correction. The volume is obtained by iterative minimisation of the following objective function:

(1) $$\underset{j}{\sum }w_{j}{\left(y_{j}^{*}-y_{j}^{s}\right)}^{2}$$

where $y_{j}^{*}$ denotes voxel intensities of scaled and bias-corrected acquired slices, $y_{j}^{s}=\sum_{i}m_{\mathrm{ij}}x_{i}$ denotes voxel intensities of slices simulated from the latest estimate of the volume with voxel intensities *x*_*i*_ using the point-spread function of MR acquisition sampled into values *m*_*ij*_. The weights *w*_*i*_ are obtained as posteriors of classification of intensity errors between simulated and corrected acquired voxels or slices into outliers and inliers using an EM algorithm (more specifically, *w*_*i*_ is a product of the voxel and slice posteriors). The reconstruction is regularized using edge-preserving smoothing (Charbonnier et al., 1997). The reconstructed volume, scaling factors and bias fields are estimated by minimising the objective function (1) using gradient descent. A high signal to noise ratio was achieved by carefully choosing the point-spread-function (PSF) to match the real acquisition. The PSF is approximated by a 3D Gaussian with full width at half maximum (FWHM) equal to slice thickness in through-plane direction to approximate the slice selection profile (truncated sinc function) and 1.2 × resolution for in-plane direction to approximate the sinc function. The combination of this comprehensive and robust reconstruction method together with high sampling and relatively small slice thickness of the acquired dataset results in a high quality fetal head volume suitable for segmentation of the structures visible in fetal US (illustrative examples are shown in Figs. 2a and 5, top row).

### 2.3 Segmentation of brain structures in MRI template

A widely used approach for segmentation of brain structures is the EM algorithm in combination with a probabilistic atlas (Leemput et al., 1999; Ashburner and Friston, 2005; Pohl et al., 2006). This approach has been also successfully used for segmentation of structures in the developing brain (Habas et al., 2010; Kuklisova-Murgasova et al., 2011a; Serag et al., 2012). In this work we assume that age-matched probability maps for six structures – WM, cortex, deep GM, brainstem, cerebellum and CSF – are available to perform EM classification.

We first upsample the reconstructed MRI into high resolution (isotropic voxels with size 0.33 mm) to obtain a high resolution segmentation. The probabilistic atlas is then aligned with the MR image and used as a spatial prior for the segmentation. The MR image is then segmented into 7 classes (white matter (WM), cortex, deep grey matter (DGM), brainstem, cerebellum, cerebro-spinal fluid (CSF) and background) using the EM algorithm (Leemput et al., 1999), by iterating between equations

(2) $$p_{\mathrm{ik}}=\frac{P\left(x_{i}^{*}|\mu_{k}\text{,}\sigma_{k}\right)p_{\mathrm{ik}}^{\mathrm{atlas}}}{\sum_{k}P\left(x_{i}^{*}|\mu_{k}\text{,}\sigma_{k}\right)p_{\mathrm{ik}}^{\mathrm{atlas}}}$$

(3) $$\mu_{k}=\frac{\sum_{i}x_{i}^{*}p_{\mathrm{ik}}}{\sum_{i}p_{\mathrm{ik}}}$$

(4) $$\sigma_{k}^{2}=\frac{\sum_{i}{\left(x_{i}^{*}-\mu_{k}\right)}^{2}p_{\mathrm{ik}}}{\sum_{i}p_{\mathrm{ik}}}$$

where $x_{i}^{*}$ denotes bias-corrected voxel intensities, *p*_*ik*_ the posteriors, and $p_{\mathrm{ik}}^{\mathrm{atlas}}$ the priors from the probabilistic atlas. $P\left(x_{i}^{*}|\mu_{k}\text{,}\sigma_{k}\right)$ are likelihoods modelled by Gaussians with means *μ*_*k*_ and variances *σ*_*k*_. The exception is the background class, where a mixture of two Gaussians is used to model the likelihood due to the presence of amniotic fluid in the background. The bias field is also iteratively corrected during segmentation. The posteriors, means and variances can be used to model the estimate of the bias-free image with voxel intensities $e_{i}=\sum_{k}\frac{p_{\mathrm{ik}}}{\sigma_{k}^{2}}\mu_{k}/\sum_{k}\frac{p_{\mathrm{ik}}}{\sigma_{k}^{2}}$ (Leemput et al., 1999). The bias field can then be estimated by comparing the image intensities to the estimate followed by weighted Gaussian smoothing (Wells III et al., 1996). Unlike previous approaches (Leemput et al., 1999; Wells III et al., 1996), we avoid a logarithmic transformation of the intensities in the pre-processing step to make the bias field additive, and work directly with a multiplicative bias field instead. At each iteration we calculate the bias residual $r_{i}=\log\left(x_{i}^{*}/e_{i}\right)$. This is followed by weighted Gaussian smoothing of the residual *r*_*i*_ with weights $x_{i}^{*}\sum_{k}\frac{p_{\mathrm{ik}}}{\sigma_{k}^{2}}$ to calculate the bias field $b_{i}^{n}$ still present at the *n*th iteration. The bias-corrected image is then updated as follows: ${\left(x_{i}^{*}\right)}^{n+1}={\left(x_{i}^{*}\right)}^{n}\exp\left(-b_{i}^{n}\right)$.

Segmentation of the brain structures is further processed to obtain the structures listed in Section 2.1. In fetal brain US, the brain tissue (with exception of the corpus callosum) is clearly divided into a right and left hemisphere. However, this is only partially visible on MRI due to its lower resolution and partial volume effects along the midline. Therefore we artificially separate the WM segmentation into two parts (by removing the voxels closer than 1.5 mm to the WM surface) and calculate distance transforms from these two eroded WM components (cores). All the brain structures (except for WM) are thus separated into right or left, depending on their distance to the right or left WM core. The distances from both cores are then regularized using Gaussian blurring (*σ* = 10 mm). Voxels with roughly the same distance from both cores (difference in regularized distances less or equal to voxel size 0.33 mm) are removed to simulate the presence of a small amount of CSF between the right and left parts of the brain, as visible in US. Since the WM-cortex boundary is not visible on fetal US, these two structures are joined to create the segmentation of cortical hemispheres.

The appearance of the cerebellum in fetal US significantly changes between 20 and 30 weeks GA (Timor-Tritsch et al., 1996; Hashimoto et al., 2001). For younger fetuses, the cerebellum does not appear as one homogeneous structure in US, but hypoechogenic WM of cerebellar hemispheres can be distinguished from hyperechogenic cerebellar cortex, see Fig. 5. By 30 weeks GA, however, the process of folding has already occurred in cerebellar hemispheres. Because this folding is of a very small scale, the cerebellum appears mainly bright on US images, with microstructure recognisable on US but not on MRI due to its lower resolution. We have therefore chosen to consider the cerebellum as a homogeneous structure for older fetuses. In younger fetuses, WM of cerebellar hemispheres has to be considered separately. We therefore manually segmented the WM of cerebellar hemispheres in the younger subject.

### 2.4 Segmentation of non-brain structures in MRI template

Some of the most echogenic landmarks in fetal US depict the non-brain structures, especially the skull, choroid plexus, septi pellucidi and membrane of the falx in the brain midline (see Figs. 2b and 5b). These structures are usually not of interest in MR studies and therefore not included in existing atlases and automatic segmentations methods. However, they play an important role in guiding the alignment of fetal brain US and MRI. We therefore performed segmentation of these four structures in MRI.

Segmentation of the skull in MRI is difficult as it appears dark and often borders other tissues also appearing dark on T2w MRI. Additionally, the fetal skull is rather thin compared to the resolution during acquisition, making it difficult to delineate due to the partial volume effect. The skull appears to closely follow the shape of the brainmask, if extra-cerebral CSF is included (see Figs. 2a and 5a). We took advantage of this fact for initial estimation of the skull segmentation in one subject. First we created the brain-mask by joining automatic segmentations of five brain tissues and CSF. A distance transform from this brain-mask was then calculated, and it was visually determined that the voxels with distance up to 2 mm could be labelled as the skull. The segmentation of the skull was then manually corrected using the manual segmentation tool provided in the IRTK package.^3^

The choroid plexus and septum pellucidum were segmented manually in one MR scan. These, as well as the skull, were then automatically transferred to new images using registration-based segmentation.

The midline voxels were estimated automatically as described in Section 2.3, and were added as another structure to simulate the falx visible on fetal brain US.

### 2.5 Automatic segmentation of unseen MRI

After segmentation of one template MRI, the segmentation of previously unseen MR images belonging to the same age-group can be performed in fully automatic manner. The template MRI is first registered to the unseen reconstructed MRI using non-rigid B-spline registration (Rueckert et al., 1999) with final control point spacing 5 mm. The segmentations of the six structures brain structures listed in Section 2.3 are transformed from the space template to the space of the new subject, blurred and used as prior probability maps for EM segmentation detailed in Section 2.3. The segmentation of non-brain structures (choroid plexus, septi pellucidi and skull) is simply transferred from the atlas using the estimated non-rigid transformation. Finally, registration-based estimation of midline is used to separate the WM into left and right hemisphere, and segmentation of falx is performed by the procedure described in Section 2.3.

### 2.6 Converting the segmentation into pseudo US image

US B-mode images are created by reflections at tissue interfaces where the two tissues differ in acoustic impedances and speckle patterns produced by interference of tissue microstructure with the sound waves. These intensity patterns are further affected by signal attenuation (or signal loss along the beam direction), shadows, which occur when a beam is fully reflected by a strong reflector, and other artefacts such as reverberations. In this work we assume that the fetal brain US is mainly composed of echogenicity of the tissues and neglect the reflections at the tissue boundaries and intensity artefacts. The visibility of the brain surface is also due to a presence of a highly echogenic thin tissue layer (Monteagudo and Timor-Tritsch, 2009) not visible in MRI. We therefore convert the segmentation to an artefact-free pseudo US image in which each region of interest is assigned a uniform intensity representing the average echogenicity of this region. As it is not possible to estimate speckle patterns from MR image, the speckle cannot be used to guide the MR-US registration. We therefore did not include a model of speckle in the pseudo US image, but rather smoothed US images using Gaussian blurring with a small kernel. The pseudo US image is then registered with a smoothed real US image using a method robust to the artefacts and missing features (Section 2.7).

Due to the complex attenuation patterns of the fetal brain US images (and its incomplete correction by the US machine), intensity and contrast of the fetal brain US images varies according to the spatial location. However, the order of brightness of different structures in a local neighbourhood is always fixed, as documented in the clinical literature (Timor-Tritsch et al., 1996; Monteagudo and Timor-Tritsch, 2009) and also observed by us. The local NCC therefore seems to be the most suitable similarity measure for matching US images. It follows that when constructing the pseudo US image, the exact value of tissue intensity is not important, but the correctness of the order of brightness is essential. The segmented anatomical structures were assigned an empirically determined intensity value in order from brightest to darkest: 1. skull, 2. choroid plexus, septi pellucidi and midline, 3. brain surface, 4. cerebellum, 5. DGM and brainstem, 6. cerebral hemispheres. The pseudo US image is shown in Fig. 4c.

### 2.7 Alignment of MRI and US

While a pseudo US image represents the ideal artefact-free US, real clinical US images are affected by attenuation and shadows, resulting in variable contrast and missing features. We therefore employ a robust block-matching strategy (Ourselin et al., 2001), which estimates a rigid or affine transformation by matching only blocks which contain features, and the similarity of the images is measured locally, between blocks, to make it independent of image contrast.

The block-matching algorithm iteratively estimates the alignment *T* between two images by alternating between two steps. In the first stage, for each block in the source image $b_{S}^{i}$, the most similar (homologous) block $b_{T}^{j}$ is found in the corresponding search neighbourhood (determined by the latest estimate of the transformation *T*) in the target image. We use NCC to find the most similar blocks, as the construction of the pseudo US image from MRI effectively changes our registration problem from multi-modal to mono-modal. NCC is calculated independently for each pair of blocks, ensuring low sensitivity to intensity artefacts. The set of vectors defined by centroids of the pairs of the homologous blocks form a displacement field which is regularized in the second stage. The blocks with variance smaller than a pre-defined threshold are excluded in the first stage.

In the second stage, rigid or affine transformation is estimated from the displacement field using least trimmed squared regression (LTS) proposed by Rousseeuw (1984):

(5) $$T^{*}=\underset{T}{\arg\min}\overset{h}{\underset{1}{\sum }}\|\left(C_{S}^{i}+d(i)\right)-T\left[C_{S}^{i}\right]{\|}^{2}$$

The LTS estimator reduces the influence of outliers by minimising the sum of a given number (*h*) of smallest squared residuals. A residual error is obtained as the difference between the displacement *d*_*i*_ at centroid $C_{S}^{i}$ and the one obtained by applying the estimated transformation to it. Such robust estimation of the transformation is essential to remove influence of displacements for the source blocks which have no corresponding target block due to missing features in the US images.

## 3 Implementation

### 3.1 The MR data and pseudo US image construction

The MR images^4^ were acquired at Hammersmith Hospital, Imperial College London, on a Philips Achieva 1.5 T scanner with parameters TR = 15,000, TE = 140–180 and excitation pulse of 90 degrees. The datasets consist of eight stacks of thin slices, with in-plane resolution 1.176 mm, the slice thickness 2.5 mm and slice overlap 1.25 mm. The images were reconstructed using the super-resolution method proposed in our previous work (Kuklisova-Murgasova et al., 2011b, 2012c) into isotropic resolution 0.75 mm and the re-oriented and re-sampled to resolution 0.33 mm. The reconstructed MRI is shown in Figs. 2a and 5, top row.

We first segmented two MRI of single subjects with GA 23 and 28 weeks, which will also serve as templates for segmenting additional MR images. The EM segmentation of brain structures was performed in a subject of 28 GA using the publicly available preterm neonatal probabilistic atlas^5^ (Kuklisova-Murgasova et al., 2011a), for which the time-point 29 weeks GA was chosen. As currently there is no fetal atlas available to us with the structures we need to segment, we used the segmentation of the older subject’s MRI as a prior for the segmentation of the younger subject’s MRI. The difference in shape and cortical folding for these GAs requires a flexible non-rigid registration and for this we used B-spline registration (Rueckert et al., 1999) with final control point spacing 2.5 mm and normalised mutual information. We found that registration of a blurry probabilistic template with such resolution is unstable, but subject-to-subject registration across such age-range produces good results for the kind of structures we are interested in. The segmentation of the older subject was transferred to the younger subject and used as a prior for the EM segmentation.

For the choroid plexus, septi pellucidi and skull, manual segmentation of these structures in one subject was needed to serve as a template for registration-based segmentation of these structures in MR scans. We manually segmented the choroid plexus and septi pellucidi in the younger subject. In the same subject, the skull was first automatically estimated using the approach presented in Section 2.4 followed by manual editing. The segmentations of these three structures were then transferred to the older subject using the B-spline registration with final control point spacing 2.5 mm. WM of cerebellar hemispheres was also manually segmented in the younger subject, but its transfer to the older subject was not necessary, as the cerebellar hemispheres do not appear hypoechogenic by 28 weeks gestation any more.

The falx was automatically segmented in both images independently using the approach described in Section 2.3.

To demonstrate the feasibility of fully automatic segmentation of unseen MRI using constructed templates, another MR image of fetal brain (22 weeks GA) was reconstructed and automatically segmented using the pipeline described in Section 2.5.

The segmentations of MR images were then converted to pseudo US images, as described in Section 2.6. An example of a pseudo US images is shown in Fig. 4c.

The complete (unoptimised for speed) segmentation and conversion pipeline takes approximately 25 min on a regular PC with Intel i7 3.4 GHz processor.

### 3.2 The US data and registration

The proposed registration method was applied to 27 US volumes of the fetal head, with GA 18–22 weeks. Additionally it was also tested on 7 US volumes with GA 28 weeks.^6^ Images were acquired with a Philips machine using a 3D transabdominal probe with mechanical steer (HD9 machine with V3-7 transducer or iU22 machine with V6-2 transducer). The acquired data were reconstructed into volumes with isotropic resolution 0.33 mm.

The images were first manually re-oriented and scaled to a similar size using a scaling factor derived from the age of the subject. The block-matching algorithm was then used to align the pseudo US image derived from the MR image with each US image. We determined the rigid alignment first, followed by the affine alignment. The block-matching was applied in two resolution levels (isotropic voxel size 2 mm and 1 mm). We used blocks of 3 × 3 × 3 voxels and neighbourhoods with 7 voxels in each direction. The variance threshold used to exclude blocks was 0.04 × intensity range in the US images, while for pseudo US images all blocks with non-zero variance were kept. The LTS transformation was estimated using 75% of the displacements. The registration of pseudo US image using block-matching method took approximately 1.25 min per subject on a regular PC with Intel i7 3.4 GHz processor, using Java implementation which has not been optimised for speed.

## 4 Results

### 4.1 Visual inspection

**Fig. 3:**
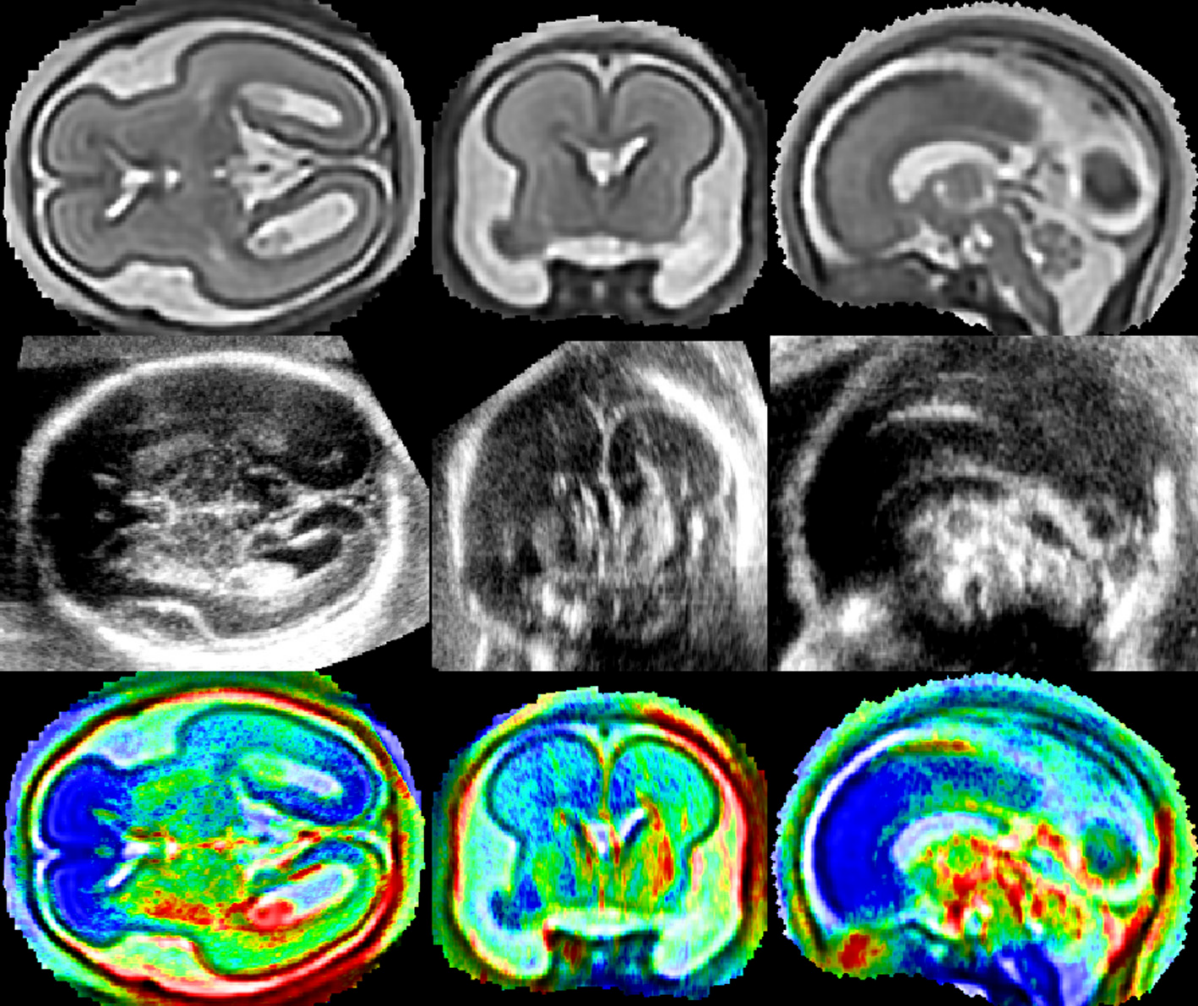
Alignment of a subject from the younger dataset with the MR template in transversal (first column), coronal (second column) and sagittal (third column) view. Top row: MR image. Middle row: US image aligned with the MR image. Bottom row: Superposition of the US image (rainbow colour-coding) over the MR image.

Registrations of all 27 US images of the younger subjects with the MR image of 23 weeks GA, as well as registrations of 7 US images of GA 29 weeks with MRI of 28 GA were visually inspected and we found that visually reasonable alignment was achieved in all cases. An example of the alignment of the younger subjects with the MRI of 23 weeks GA is shown in Fig. 3.

**Fig. 4:**
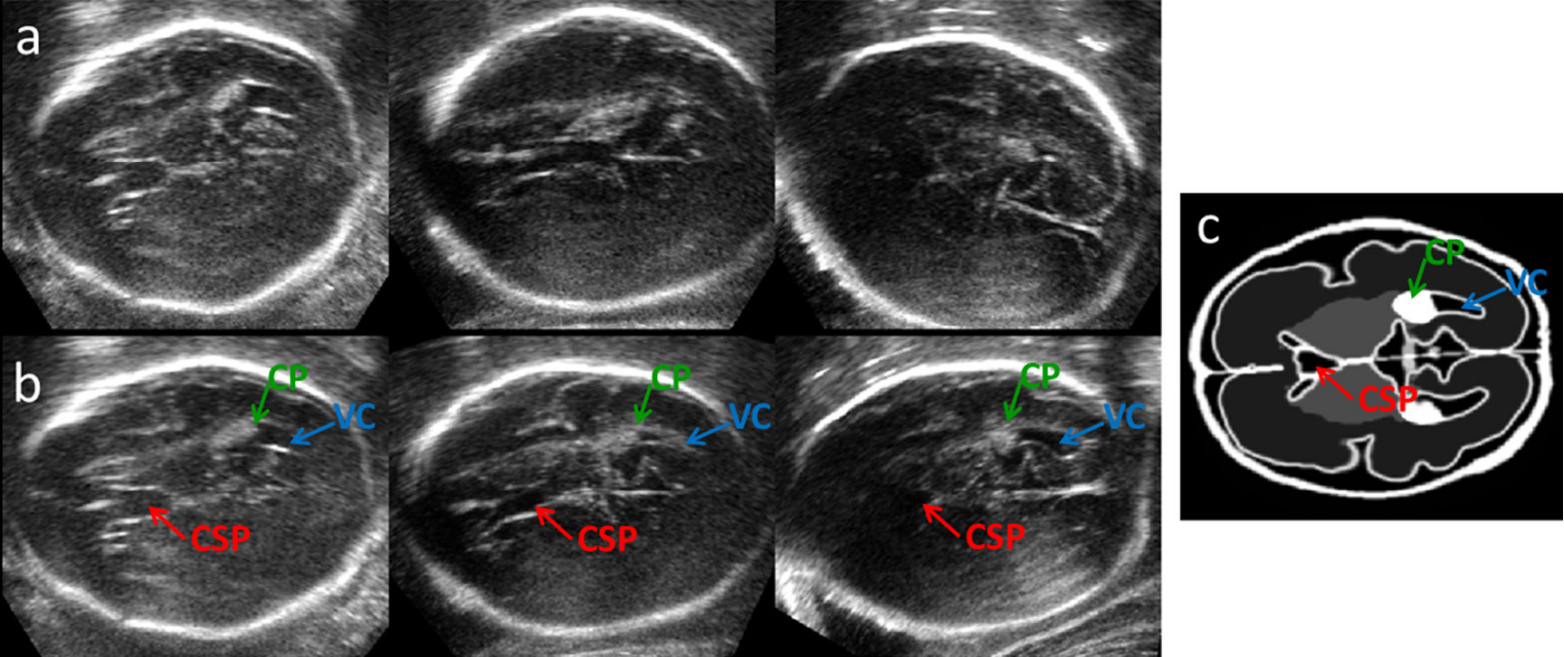
A 2D view of 3D US of three fetuses with GA around 29 weeks before (a) and after (b) alignment with the MRI. After alignment the view shows the transventricular plane in all three cases. Compare to pseudo US image (c). Note that CP, CSP and VC are visible, while cerebellum does not appear, as expected in transventricular plane.

**Fig. 5:**
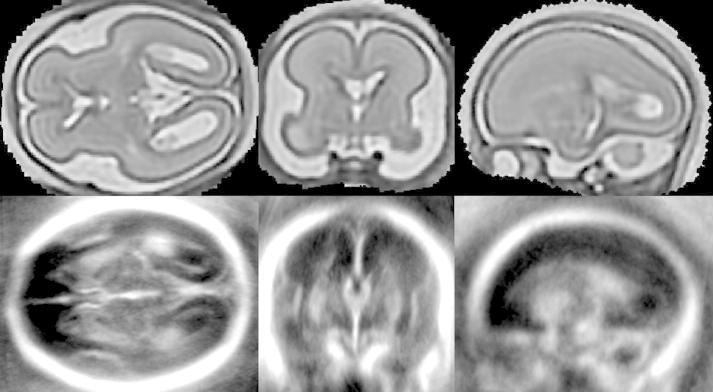
Axial, coronal and sagittal view of brain MRI of a fetus with GA 23 weeks (first row) and the average of the 27 fetal brain 3D US with GA 18–22 weeks, aligned with the MRI.

The alignment results of three US images with GA 29 weeks with MRI with 28 GA are presented in Fig. 4. The figure depicts the transventricular plane (ISUOG, 2007) in the pseudo US image (Fig. 3c). This plane is often selected for clinical assessment of fetal brain development. While before alignment the individual US volumes show variable features in this plane (Fig. 3a), after the alignment all appear to show the transventricular plane. In this plane CP, CSP and VC should be visible, while cerebellum should not appear in this view (ISUOG, 2007). To further illustrate the quality of alignment, Fig. 5 presents the average of all 27 US volumes from the younger dataset aligned with the younger MR template using the estimated transformations.

### 4.2 Qualitative evaluation of the younger dataset alignment

Alignment of the 27 US images with GA 18–22 weeks with the MR image with GA 23 weeks is a challenging task, as the size and shape of the brain changes rapidly at this GA. Example of such change is deepening of the Sylvian fissure. We therefore performed a detailed qualitative assessment of the affine alignment of these US volumes with the MRI.

A senior neuroradiologist (GQ) and a senior obstetrician (AP), evaluated the alignment. Six landmarks were chosen for qualitative assessment, see Table 1. The clinicians assessed alignment of each landmark by assigning them into one of the four categories: Good, Acceptable, Unacceptable or Not visible. The decision was reached by consensus agreement between both experts. The qualitative results for Corpus Callosum, Cerebellar Hemisphere and Cavum Septum Pellucidum were excellent, with all except one subject showing good alignment when the landmark was visible. The alignment for the remaining one subject (which was different for each landmark) was assessed as acceptable. The Sylvian fissure and anterior lateral ventricle scored slightly less well, with 75% and 81% of subjects having good alignment. These two structures are most affected by shape and location change between 18 and 23 weeks GA. In most cases, the MR image well predicted the location of Sylvian fissure in the US subject at 23 weeks GA, in opinion of both experts. The performance of the method was overall judged as good by both experts.

## 5 Quantitative evaluation

The traditional approach for quantitative assessment of the alignment is to use manually placed landmarks – the anatomical location points which can be reliably identified in both source and target image – and calculating the distance of these landmarks after alignment. Such strategy has been also used in some of the CT/MR-US registration works (e.g. Arbel et al., 2004; Wein et al., 2008). In the brain imaging community, however, the landmark based evaluation approach is less frequently used due to the difficulties in unambiguously defining anatomical landmarks on the complex shape of the 3D brain. Consequently surrogate measures, such as tissue/structure overlaps or similarity measures are commonly used. A recent paper by Rohlfing (2012) questioned the reliability of these measures and concluded that only volume overlaps of localised anatomical structures provide a reliable surrogate measure of registration accuracy, as they can serve as an approximation of the landmark-based approach when the anatomical landmark points cannot be reliably identified.

Reliable identification of anatomical locations in 3D US images of fetal brain is further complicated by artefacts resulting from shadows, reverberations and blurring caused by the anisotropic point-spread function. Because of these limitations, four small regions which can be relatively well identified in the fetal brain US images were chosen and manually delineated in the younger dataset (around 20 weeks GA), and their overlaps with the MRI segmentation were used for evaluation. In the older dataset (around 29 weeks GA) such segmentations were not available, and we therefore placed five landmarks in both the US and MR images of the older dataset to quantitatively evaluate the alignment performance. While the regional overlaps in the younger dataset showed adequate sensitivity to distinguish between the performances of the different alignment methods investigated, for the landmark-based approach used for the older dataset the intra-rater variability of manual placement was not statistically significantly different from differences in the automatic (performed by affine alignment and transferring the landmarks from MRI) and manual landmark placement.

### 5.1 Evaluation of younger dataset alignment

In each of the 27 ultrasound volumes, four small structures – choroid plexus (CP), cavum septi pellucidi (CSP), posterior ventricular cavity (VC) and cerebellar hemisphere (CH) – were manually delineated by a clinical expert (RN). All these structures were also delineated in the MR image. The average size of these structures as estimated from the manual segmentations ranges between 0.2 and 1.1 cm^3^ after transfer to the space of the MR template (see Table 2, first row). This can be compared to the size of the cranial cavity of the MR template, with volume of 194 cm^3^. These structures are therefore sufficiently localised for evaluation of registration accuracy. Due to the small size of these structures, the low flexibility of the affine transformation, and inter-subject registration problem, we do not expect high Dice overlaps even for high quality alignment.

To estimate the maximal possible average overlap for each of these structures, we registered the manual segmentations of the US images with the manual segmentation of the MR template using label consistency as a similarity measure and gradient descent as the optimisation method. The registrations were initialised with the transformations estimated using the proposed method to ensure full success rate of the gradient descent optimisation. The second row of Table 2 shows that the estimated maximum expected average Dice overlaps range from 0.4 to 0.66. The third row of Table 2 shows the Dice overlaps for our proposed method, ranging from 0.43 to 0.58. We can conclude that the performance of our proposed method reaches the estimated maximum possible performance, by being very similar or even better for three out of four structures, with some margin for improvement for the choroid plexus. For all tested US scans, all four structures were overlapping after the alignment using our proposed method. We can therefore conclude, given the very small size of the delineated structures, that none of the 27 registrations failed.

We also present another measure of accuracy of the registration to quantitatively evaluate the geometric error. We calculated the barycentres of the manual segmentations to approximate manual placement of landmarks. Distances of these barycentres give an estimate of the target registration error (TRE). We perform a similar experiment as for Dice overlaps to estimate the lower bound of the TRE. The fourth and fifth row of Table 2 state the TRE for label-consistency and our proposed method, respectively. While with label consistency we achieved a mean TRE (mTRE) over the four landmarks of 2.04 mm, the proposed method gives only a slightly larger mTRE of 2.36 mm.

**Fig. 6:**
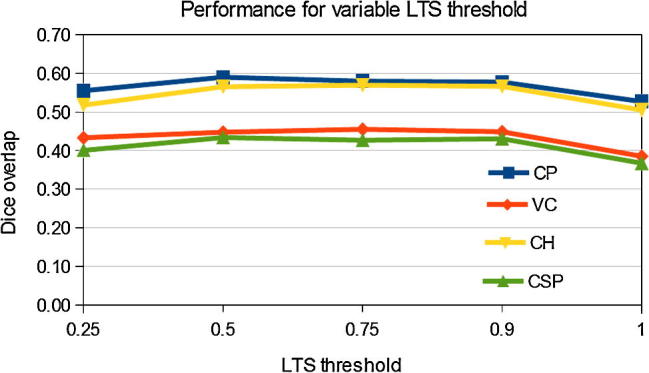
Average dice overlaps of manual segmentation of structures in the MR template and 27 US images in the younger dataset, depending on the threshold used during LTS optimisation.

We further investigated the influence of the block-matching parameters on the performance of the method. In our experiments we found that varying the block-matching parameters resulted in only a very minor variation of the performance. The influence of the value for the threshold in the LTS optimisation on the performance is presented in Fig. 6. Varying the LTS threshold resulted in no statistically significant differences in performance if kept within reasonable bounds, e.g. between 0.5 and 0.9. Setting this threshold to value 1 (which is equivalent to replacing robust LTS with standard least square optimisation), resulted in a drop in performance, which was statistically significant for three out of four structures and one of the registrations failed (three out of four structures had a zero overlap). This suggests that excluding outlier displacement is essential for achieving a good performance for the method. We found that excluding blocks with small variance did not result in statistically significant improvement of the performance when the chosen threshold 0.75 was used for LTS. This is most likely due to the effectiveness of the robust LTS optimisation, which seems to be sufficient for exclusion of inconsistent displacements assigned to blocks with missing features. Increasing the size of the blocks from our chosen size 3 × 3 × 3 to values 5 × 5 × 5 or 7 × 7 × 7 did not result in statistically significant differences in performance, however the average Dice overlaps dropped slightly for three out of four structures and the computational time increased. These results show the stability of the registration method with respect to the block-matching parameters and confirm the suitability of the parameter choice as presented in Section 3.2.

### 5.2 Quantitative comparison with direct multi-modal and other pseudo US image based registration methods

To demonstrate that converting MRI into a pseudo US image is essential for good alignment, we compared our proposed method with two recognised approaches that can perform multi-modal registration of the original MRI with US volumes: Registration using normalised mutual information (NMI); and th block-matching method using correlation ratio (CR). NMI (Studholme et al., 1999) is a statistical similarity measure based on the normalised entropy of the joint intensity histogram of the two images and is widely accepted as a suitable multi-modal similarity measure for various applications. In our experiments we used the registration tool implemented in IRTK,^7^ where rigid and affine transformations are found using gradient descent optimisation and NMI as a similarity measure. The multimodal block-matching method with CR as similarity measure was proposed in the original paper describing the block-matching algorithm by Ourselin et al. (2001). When using CR as a similarity measure, the functional relationship between intensities of the target and source image can be implemented by a statistical (non-parametric) or parametric model. Due to the small size of the blocks in our approach, the parametric affine relationship of the intensities can be considered as a reasonable assumption. In this case CR is equivalent to squared NCC.

Additionally, we compare the proposed block-matching registration of the pseudo US image with gradient descent optimisation using local normalised cross-correlation (LNCC) or global normalised cross-correlation (NCC), to demonstrate that block-matching alignment with robust LTS optimisation plays an essential role in achieving good performance when applied to alignment of fetal brain US and MRI. Gradient descent based registration using NCC and LNCC were previously utilised in works of Arbel et al. (2004) and Mercier et al. (2012) to align a pseudo US image with US of the adult brain. It is important to point out though, that the method of Arbel et al. (2004) was used for non-rigid alignment of the brain-shift during neurosurgery and included a directional gradient component, so it cannot be directly compared to the methods considered here.

The average volume overlaps of the segmented structures for each tested alignment method are presented in Table 3. We also present the statistical significance of differences in average overlaps compared to the proposed method, calculated using paired *t*-test. All the tested methods improved the dice overlaps compared to the intitial alignment. In the first multimodal approach (NMI and gradient descent) the average volume overlaps were much smaller than for our proposed method. The performance was boosted when the pseudo US image and LNCC or NCC were used. However, the performance using both similarity measures was significantly below the proposed method, measured by both average Dice overlaps and by *t*-test. All tested gradient descent approaches were outperformed using block-matching methods. In the multimodal block-matching approach the average overlaps were smaller than for our proposed method and these differences were statistically significant for three out of four structures. Statistical significance was not proven for the posterior ventricular cavity, the structure with the largest variance in overlap across subjects, which is very often poorly visible in the US images of younger subjects. In the last experiment we compared the performance of NCC and CR when registering the pseudo US image with real US volumes. Mono-modal NCC yields better performance for the block-matching of the pseudo US image with real US images than the less constrained multimodal CR, with statistically significant improvement for two out of four structures, though the overall improvement was only marginal. These results demonstrate that converting MRI into a pseudo US image and using locally adaptable monomodal similarity measure offers better correspondences for estimation of a good alignment compared to the direct multimodal MR/US case.

**Fig. 7:**
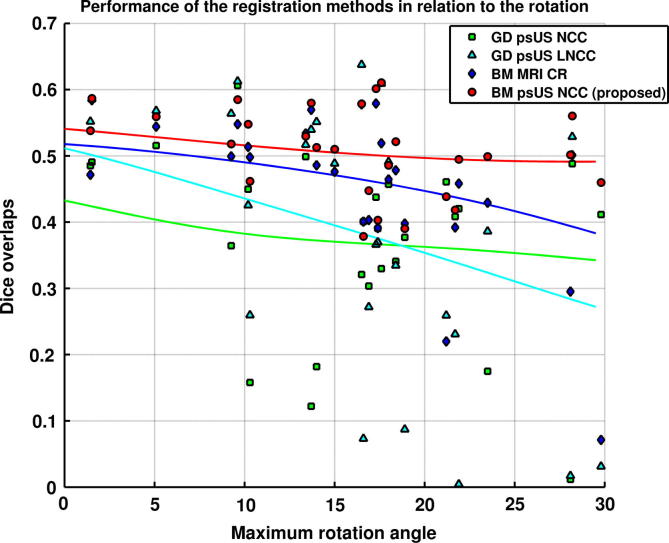
Registration of 27 US images of younger dataset with MR template. Performance of the four methods in relation to the maximum Euler rotation angle that needs to be recovered for correct alignment. Each point represents average dice overlap for four structures in a single subject. We also show a trendline for each of the four methods, calculated using Gaussian kernel regression (*σ* = 7.5°).

Fig. 7 presents a scatter-plot that relates average Dice overlaps over all four structures against the maximum Euler rotation angle that needs to be recovered to align US images to MRI. This plot indicates that the larger rotations are a significant factor for the worse performance of the other methods compared to the proposed method, showing that one of the main advantages of our method lies in its extended robustness towards unfavourable initialisation. The trend lines are also included in Fig. 7 o show patterns of performance of the different methods. For the proposed method, the performance is largely sustained with rotations of up to 30°. Multimodal block-matching performs poorly for several subjects for which larger rotations need to be recovered. The gradient descent registration results in a significant number of poorly aligned images for rotations higher than 10°.

Table 4 presents the comparison similar to the Table 3, except that we here calculate the distance of the barycentres of manual segmentations. We can observe that patterns of performance are similar to the results obtained using Dice overlaps, which clearly confirms superior performance of block-matching compared to the gradient-descent based methods. However, the proposed method achieved statistically significant improvement for only two out of four structures compared to multimodal block-matching. Exchanging NCC for CR when pseudo US based block-matching is used results in significantly reduced performance only for one structure, indicating that the choice between the two similarity measures is the least important factor contributing to the quality of the registration for the pseudo US image based block-matching.

### 5.3 Influence of fully automatic segmentation of MRI on registration performance

In Section 2.5 we described a fully automated pipeline for segmentation of unseen fetal MRI using the MR template described in Sections 2.3 and 2.4. In this section we investigate whether the performance of the proposed registration method can be sustained when an unseen MRI is to be aligned with US images, rather than the original template, using four unseen MR images of fetuses with various GA, ranging from 20 to 23 weeks. We compare the proposed method to three other methods, namely a multimodal block-matching algorithm, and pseudo US imaged based gradient descent algorithms using NCC and LNCC as similarity measures. This experiment also offers more extensive evaluation of the performance of the tested methods, as we register each of the 27 US images in the younger dataset to each of the four unseen MR images, with 108 registrations in total. We calculated average Dice overlaps for each of the four structures as well as the average distance of barycentres of the manual segmentations for these four structures, over all 108 registrations. The results presented in Tables 5,6 show better performance of the proposed method using both measures compared to other three tested methods. The increase in performance is statistically significant, with exception of the ventricular cavity for multimodal and pseudo US based block-matching. We can also observe that average Dice overlaps for our proposed method are still in a similar range (0.40–0.56) as when using the original MR template. Similarly, the distance of barycentres of the manual segmentations (mTRE 2.52 mm), is still close to the lower bound of 2.04 mm, see Table 2. Though registration error and anatomical variability cannot be decoupled in inter-subject registration (especially in case of affine registration), these results were obtained using variety of anatomies and ages (within 18–24 weeks GA interval), indicating that the basic pattern of performance for the four tested methods does not change with normal variation of fetal brain anatomy. These experiments demonstrate the feasibility of the proposed method for the alignment of unseen MRI and US fetal brain image volumes.

### 5.4 Evaluation of the older dataset alignment

In each of the seven US images in the older dataset (29 weeks GA) and the MR template of 28 weeks GA, five landmarks were placed under supervision of a clinical expert (GQ). The chosen landmarks were: 1. Posterior wall of cavum septi pellucidi (CSP); 2. Fourth ventricle (FV); 3. Sylvian fissure (SF); 4. Temporal horn of lateral ventricle (THLV); 5. Occipital horn of lateral ventricle (OHLV). Since the placement of landmarks was found to be very difficult in 3D US, they were placed twice to estimate the intra-rater error. The landmarks were also placed in the MR template, which was registered using our proposed method to all seven 3D US images. Landmarks from the MR image were then propagated to the US images using the estimated affine transformation (referred to as automatic landmark placement). The mean target registration error (mTRE), calculated as average distance of the five landmarks in US and MRI after the alignment was used for evaluation.

The results of this experiment are presented in Table 7. The mTRE for the proposed method was 2.52 mm, slightly larger than the intra-rater mTRE at 2.14 mm. Although the absolute mTRE value of the intra-rater error is smaller than the registration error for the proposed method, the paired two-tailed *t*-test did not show statistically significant difference between registration and intra-rater error, with a large *p*-value of 0.81. This is probably due to the large standard deviation of the intra-rater error. We therefore conclude that landmark placement in 3D US of the fetal brain is not more reliable than the proposed automatic registration, as placing them automatically using our proposed method performs comparably to a manual rater. However, this result also shows that the proposed method succeeded in alignment of the seven 3D US scans of fetal brain with MRI.

We also compared the proposed method to the three additional methods (multi-modal blockmatching and pseudo US based gradient descent registration using NCC and LNCC), see Table 7. The mTRE was higher in all three methods compared to the proposed method, with the drop in performance of the gradient-descent based methods being statistically significant. Statistical significance of the difference between multimodal and pseudo US image based block-matching was not proven (*p* = 0.15), however, the further decrease in performance in multi-modal blockmatching compared to the proposed method meant, that automatic landmark placement using multi-modal blockmatching was not comparable to the manual rater any more (*p* = 0.03). Thus only the proposed method was as successful in automatically placing the landmarks as the manual rater.

## 6 Discussion

The methodology described in this paper was developed with anticipation of 3D US and multi-modal studies of fetal brain development. The major drawback of 3D US as a modality for phenotypical description of the fetal brain is incompleteness of the image, as position of the fetus is not always ideal for scanning and field of view is always at least partially restricted by fetal skull. Therefore template images of fetal brain built from MRI, such as pseudo US image presented in this paper, can be very valuable in a sense that they offer models of complete fetal brain anatomy, unlike a single 3D US scan. Such templates can thus offer correspondences for any US image independent of the position of the probe, shadows and missing anatomical regions. In this paper we demonstrated that pseudo US image constructed from fetal MRI can be successfully used for co-alignment of a set of US images, an important initial step for further processing of the medical images. The average of these 3D US images in turn revealed, that 3D US can depict the fetal brain anatomy in great detail, highlighting the potential of this modality in large population studies, such as for phenotypical comparison of normal and growth-restricted fetal populations, in spite of missing anatomical features in any single 3D US scan.

The method thus can be readily used for the alignment of the fetal brain US images into the standard orientation, which can assist visual interpretation of the US. Automatic orientation of 3D US can also assist in clinical 2D measurements by automatically determining the biometric plane, which is normally manually selected by the sonographer during 2D acquisition.

The affine transformation of the US image to the MR template can be also used for estimating the head volume, a 3D equivalent of routine 2D measurements such as head diameter and circumference, which heavily depend on the correct selection of the biometry plane.

**Fig. 8:**
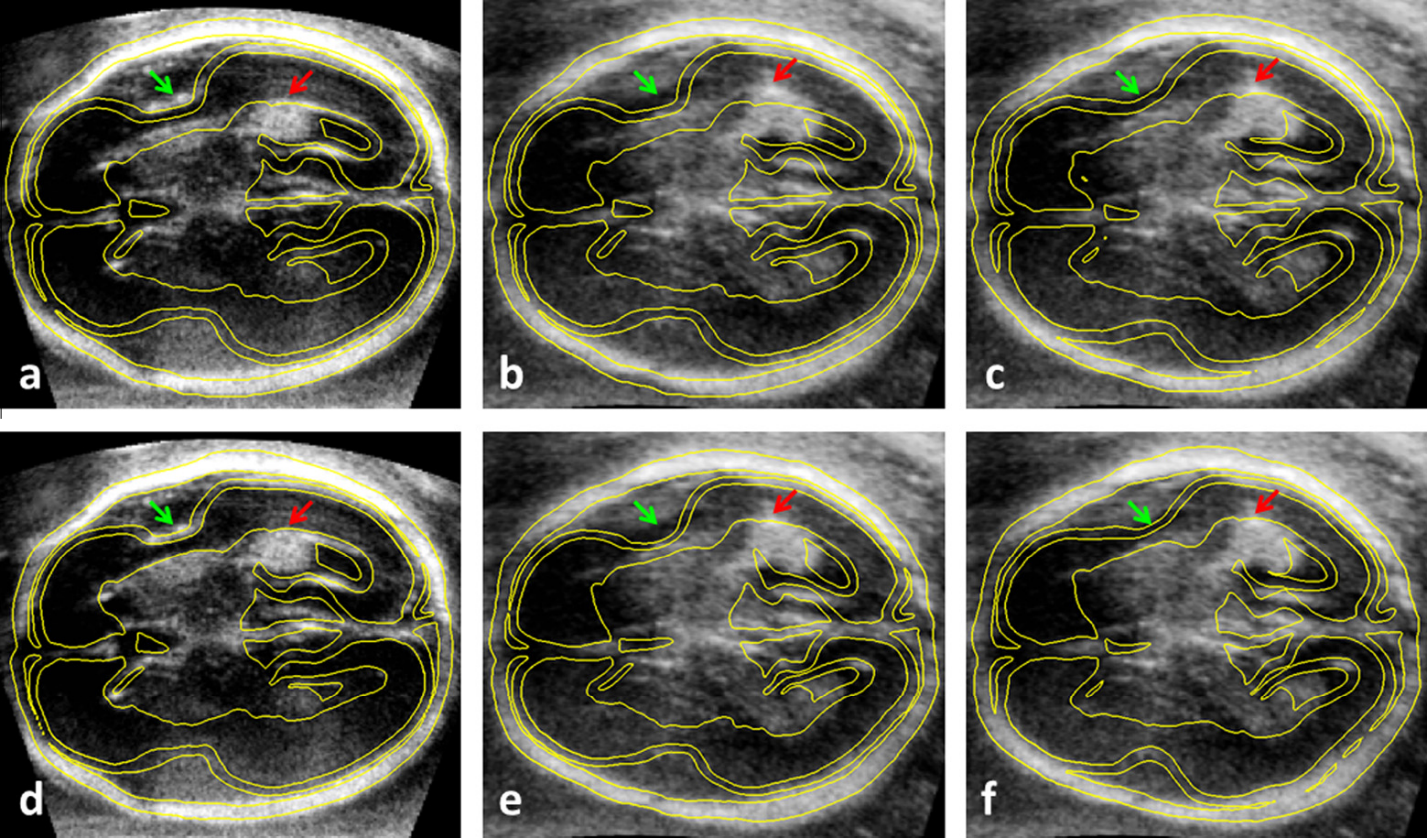
Ultrasound images of two fetuses of different GA registered to the MR template, with superimposed isolines of the pseudo US image simulated from MRI. The older fetus (approximatelly 22 weeks GA) is presented in (a) and (d) and the younger fetus (approximately 18 weeks GA) in (b), (c), (e) and (f). The pseudo US was simulated from MRI of a subject with (a,b) 23 weeks GA and affinely aligned with the US; (d,e) 23 weeks GA and non-linearly aligned with the US; (c) 20 weeks GA and affinely aligned with the US; (f) 20 weeks GA and non-linearly aligned with the US. The arrows point to features indicative of brain maturity, namely Sylvian fissure (green) and Choroid plexus (red). The images illustrate that choosing a template that matches the age of the subject more closely (c) can be as effective for alignment of these features as non-linear registration (e). The non-linear registration might improve feature alignment for MR/US of the similar ages, as shown in (d). However, when features are not clearly visible, the non-linear registration might not be an effective solution to the aligment problem, such as for Sylvian fissure of the younger fetus shown by the green arrow in (e) and to a lesser extent in (f). (For interpretation of the references to colour in this figure legend, the reader is referred to the web version of this article.)

When looking for biomarkers of healthy brain development, size is not always the best indicator of normal brain growth. The affine co-alignment of 3D US images with an atlas of the normal brain anatomy can thus form a basis for finding size-independent US-based biomarkers of the brain maturity. In Fig. 8b,c we demonstrate that choosing a template of a similar age results in improved alignment of the brain features after affine registration. This indicates that an age-matched template might be suitable for identifying deviations from normal development. As more high quality fetal MRI of young fetuses become available, we will be able to construct a spatio-temporal pseudo US template of normal brain development. By looking at features such as the deepening Sylvian fissure (highlighted by the green arrow in Fig. 8), such an atlas could provide a tool for 3D US-based identification of developmental delay, intra-uterine growth restrictions or other conditions affecting brain growth.

The quantitative landmark-based evaluation of age-matched dataset presented in Section 5.4 revealed that the proposed method was as good in automatically placing the landmarks in 3D US by propagation from MRI as the manual rater. Though this result reflects good performance of the proposed alignment method, it also shows how difficult the task of navigation of fetal US by sonographer can be, thus highlighting the potential benefit of automatic localisation of structures in the fetal brain, which could be achieved using age-matched MR template. When considering tasks such as anatomical localisation or navigation, it might be beneficial to develop methodology for non-linear alignment of the pseudo US image with 3D US. However, increasing degrees of freedom for the spatial alignment also increases the susceptibility of the method for overfitting due to the imaging artefacts and missing features, making a design of robust non-linear alignment methodology a very challenging task. Fig. 8d-f shows a result for non-linear alignment of a pseudo US template with a 3D US using a locally adaptive extension of the block-matching algorithm (Pitiot and Guimond, 2008). In this example we used a radius of 3 mm to calculate a locally affine transformation. Though the alignment of the features visibly improved, using a more closely age-matched affinely registered template can have similar effect to non-linear registration (compare Fig. 8c and e). Additionally non-linear registration is not an effective solution in areas where features are not clearly visible, such as Sylvian fissure of the younger fetus (see Fig. 8e and f). To make a more flexible non-linear registration feasible, it might be beneficial to improve on the imaging techniques for 3D US of the fetal brain, in order to minimise the shadows in the image, for example by scanning through the fontanel. Additionally, non-linear registration methodology could be used to improve the alignment with the age-matched atlas, as demonstrated in Fig. 8d, as this would allow the registration to be less susceptible to misalignment due to missing features and artefacts.

Another possible application of the proposed methodology is intra-subject MR-US rigid registration. We demonstrated that an unseen fetal brain MRI can be segmented using the constructed MR template and segmentation further converted to the pseudo US image. MR and US of the same subject can thus be aligned using a fully automatic pipeline. The intra-subject MR-US alignment tool can be used in applications such as detailed comparison of the features in both modalities, to determine whether fusion of infor mation from fetal brain MRI and US could enhance abnormality screening. As manual measurements obtained from 3D US exhibit rather high degree of uncertainty, intra-subject MR-US alignment can also facilitate use of MRI for validation of methods for automatic image analysis of 3D US, such as quantitative measurements, identification of key fetal structures and finding biometry planes.

It remains for the future work to establish the role of the presented work in clinical practice, such as making the alignment real-time, accuracy needs to be established for different potential tasks, such as detection, localisation or quantification, and clinical studies need to be conducted.

## 7 Conclusion

In this paper we have presented a novel method for rigid or affine registration of fetal brain MR and US volumes. The method was successfully applied to affinely align US volumes with an age-matched MRI at two different time-points of gestation. In our experiments we achieved good qualitative results as well as volume overlaps for four small structures. The average of the co-aligned US volumes revealed near-complete anatomy of the fetal brain. Our results suggest that 3D US in conjunction with an MR prior has potential for enhancement of ultrasonic visualisation of fetal brain anatomy. The proposed registration tool can now facilitate utilisation of models of fetal brain anatomy extracted from MRI to enhance image analysis of fetal brain 3D US. Good multi-modal alignment between MRI and US can also facilitate validation of automatic image analysis of 3D US using MRI and fusion of information from both modalities.

## Figures and Tables

**Table 1:** Qualitative assessment of MR-US alignment of six landmarks (Column 1) as observed in one of the standard views (Column 2). The table shows the number of subjects with quality of alignment for each landmark assigned to one of the four categories (Columns 3–6).

Structure	View	Good	Acceptable	Unacceptable	Not visible
Corpus callosum	Sagittal	23	1	0	3
Cerebellar hemisphere	Sagittal	26	1	0	0
Cerebellar hemisphere	Axial	26	1	0	0
Cavum septum pellucidum	Axial	26	1	0	0
Sylvian fissure	Coronal	18	6	0	3
Anterior lateral ventricle	Coronal	22	4	1	0

**Table 2:** Evaluation of alignment using manual delineation of four small structures. The first row shows volumes of these structures. Compare to the volume of the cranial cavity 194 cm^3^. The second row shows the average Dice overlaps and their standard deviations after registration of manual segmentations of these structures using label consistency as a similarity measure. The third row shows Dice overlaps after alignment using our proposed method. The fourth and fifth row show distance of barycentres of manual segmentations after alignment using label consistency and the proposed method, respectively.

	CP	VC	CH	CSP	Average
Structure volume	1.10 cm^3^	0.35 cm^3^	0.41 cm^3^	0.19 cm^3^	0.51 cm^3^
Dice overlaps using label consistency	0.66 ± 0.07	0.47 ± 0.17	0.61 ± 0.11	0.40 ± 0.16	0.54 ± 0.13
Dice overlaps using proposed method	0.58 ± 0.10	0.46 ± 0.15	0.57 ± 0.09	0.43 ± 0.10	0.51 ± 0.11
Distance of barycentres using label consistency	1.76 ± 0.95	1.98 ± 1.25	1.59 ± 0.77	2.83 ± 1.24	2.04 ± 1.05
Distance of barycentres using proposed method	2.38 ± 1.06	2.42 ± 1.30	1.87 ± 0.70	2.77 ± 1.40	2.36 ± 1.11

**Table 3:** Registration of 27 US images of younger dataset with MR template. Average volume overlaps of four manually segmented structures and their standard deviations after registration of the source image (third column) to the US images. We also present *p*-value showing statistical significance when compared to the proposed method. The best performance is highlighted in bold.

Registration	Similarity	Source	CP	VC	CH	CSP	Average
No alignment			0.18 ± 0.14	0.10 ± 0.15	0.09 ± 0.13	0.09 ± 0.14	0.12 ± 0.14
Gradient descent	NMI	MRI	0.28 ± 0.19	0.18 ± 0.18	0.27 ± 0.19	0.22 ± 0.17	0.24 ± 0.18
*p*-value			1 × 10^−8^	2 × 10^−9^	2 × 10^−7^	3 × 10^−6^	6 × 10^−12^
Gradient descent	NCC	Pseudo US	0.45 ± 0.16	0.34 ± 0.19	0.31 ± 0.20	0.40 ± 0.17	0.37 ± 0.18
*p*-value			2 × 10^−4^	0.022	1 × 10^−6^	0.21	7 × 10^−5^
Gradient descent	LNCC	Pseudo US	0.42 ± 0.25	0.35 ± 0.26	0.45 ± 0.25	0.32 ± 0.18	0.38 ± 0.23
*p*-value			3 × 10^−4^	1 × 10^−3^	0.021	4 × 10^−3^	5 × 10^−4^
Block-matching	CR	MRI	0.54 ± 0.13	0.43 ± 0.17	0.46 ± 0.15	0.39 ± 0.13	0.46 ± 0.14
*p*-value			0.048	0.30	3 × 10^−4^	6 × 10^−3^	5 × 10^−3^
Block-matching	CR	Pseudo US	**0.58** ± 0.11	0.42 ± 0.17	0.54 ± 0.16	0.39 ± 0.11	0.48 ± 0.14
*p*-value			0.92	0.043	0.31	0.010	0.13
Block-matching	NCC	Pseudo US	**0.58** ± 0.10	**0.46** ± 0.15	**0.57** ± 0.09	**0.43** ± 0.10	**0.51** ± 0.11

**Table 4:** Registration of 27 US images of younger dataset with MR template. Average distance of barycentres (in mm) of the four manually segmented structures and their standard deviations after registration of the source image (third column) to the US images. We also present *p*-value showing statistical significance when compared to the proposed method.

Registration	Similarity	Source	CP	VC	CH	CSP	Average
No alignment			6.50 ± 2.65	7.75 ± 3.43	7.15 ± 2.52	7.37 ± 2.75	7.19 ± 2.84
Gradient descent	NMI	MRI	5.66 ± 3.19	6.54 ± 3.85	5.68 ± 4.37	4.80 ± 2.46	5.67 ± 3.47
*p*-value			4 × 10^−6^	7 × 10^−6^	2 × 10^−4^	6 × 10^−5^	1 × 10^−6^
Gradient descent	NCC	Pseudo US	3.51 ± 2.06	3.68 ± 2.86	4.67 ± 2.87	3.20 ± 2.24	3.76 ± 2.51
*p*-value			6 × 10^−3^	0.041	3 × 10^−5^	0.32	1 × 10^−3^
Gradient descent	LNCC	Pseudo US	4.33 ± 2.95	4.69 ± 4.13	3.36 ± 3.41	3.87 ± 2.36	4.06 ± 3.21
*p*-value			5 × 10^−4^	4 × 10^−3^	0.033	0.011	2 × 10^−3^
Block-matching	CR	MRI	2.79 ± 1.57	2.74 ± 01.58	2.95 ± 1.91	3.54 ± 2.14	3.00 ± 1.80
*p*-value			0.077	0.28	3 × 10^−3^	5 × 10^−3^	0.02
Block-matching	CR	Pseudo US	2.45 ± 1.29	2.82 ± 1.71	2.19 ± 1.56	3.11 ± 1.58	2.64 ± 1.54
*p*-value			0.63	0.089	0.25	4 × 10^−3^	0.11
Block-matching	NCC	Pseudo US	**2.38** ± 1.06	**2.42** ± 1.30	**1.87** ± 0.70	**2.77** ± 1.40	**2.36** ± 1.11

**Table 5:** Registration of 27 US images of younger dataset with four unseen MR subjects. Average volume overlaps of four manually segmented structures and their standard deviations after registration of the source images (third column) to the US images. We also present *p*-value showing statistical significance when compared to the proposed method.

Registration	Similarity	Source	CP	VC	CH	CSP	Average
No alignment			0.15 ± 0.14	0.08 ± 0.12	0.07 ± 0.11	0.07 ± 0.12	0.09 ± 0.07
Gradient descent	NCC	Pseudo US	0.41 ± 0.15	0.33 ± 0.15	0.35 ± 0.16	0.37 ± 0.16	0.37 ± 0.10
*p*-value			5 × 10^−25^	1 × 10^−7^	2 × 10^−20^	0.01	1 × 10^−22^
Gradient descent	LNCC	Pseudo US	0.33 ± 0.22	0.29 ± 0.19	0.35 ± 0.23	0.29 ± 0.17	0.31 ± 0.17
*p*-value			3 × 10^−20^	1 × 10^−13^	3 × 10^−11^	2 × 10^−12^	2 × 10^−18^
Block-matching	CR	MRI	0.51 ± 0.13	0.42 ± 0.15	0.48 ± 0.12	0.38 ± 0.13	0.45 ± 0.09
*p*-value			2 × 10^−8^	0.09	6 × 10^−3^	4 × 10^−5^	9 × 10^−6^
Block-matching	NCC	Pseudo US	**0.56** ± 0.11	**0.43** ± 0.14	**0.51** ± 0.09	**0.40** ± 0.10	**0.48** ± 0.06

**Table 6:** Registration of 27 US images of younger dataset with four unseen MR subjects. Average distance of barycentres (in mm) of the four manually segmented structures and their standard deviations after registration of the source images (third column) to the US images. We also present *p*-value showing statistical significance when compared to the proposed method.

Registration	Similarity	Source	CP	VC	CH	CSP	Average
No alignment			7.12 ± 2.88	7.87 ± 3.29	7.55 ± 2.55	7.65 ± 2.91	7.55 ± 1.92
Gradient descent	NCC	Pseudo US	3.49 ± 1.41	3.25 ± 1.47	3.24 ± 1.47	3.47 ± 2.81	3.36 ± 1.26
*p*-value			1 × 10^−14^	8 × 10^−3^	1 × 10^−13^	0.03	4 × 10^−12^
Gradient descent	LNCC	Pseudo US	4.89 ± 2.76	4.78 ± 3.43	3.92 ± 3.37	4.31 ± 2.93	4.48 ± 2.5
*p*-value			3 × 10^−20^	1 × 10^−13^	3 × 10^−11^	2 × 10^−12^	2 × 10^−18^
Block-matching	CR	MRI	2.82 ± 1.34	2.97 ± 1.68	2.33 ± 1.41	3.35 ± 1.62	2.87 ± 0.98
*p*-value			1 × 10^−5^	0.25	2 × 10^−3^	3 × 10^−9^	6 × 10^−5^
Block-matching	NCC	Pseudo US	**2.37** ± 1.11	**2.83** ± 1.42	**1.95** ± 0.76	**2.93** ± 1.46	**2.52** ± 0.54

**Table 7:** Evaluation of the alignment using five manually placed landmarks. The landmarks were place twice in US images and the last row shows the intra-rater error. The mTRE is calculated over the five landmarks for each of the tested methods. In the last two columns we present *p*-values showing the statistical significance when compared to the manual rater and the proposed method, respectively.

Registration	Similarity	Source	mTRE (mm)	*p*-value with intra-rater	*p*-value with proposed
No alignment			7.49	6 × 10^−4^	6 × 10^−4^
Gradient descent	NCC	Pseudo US	3.18	0.03	0.03
Gradient descent	LNCC	Pseudo US	4.73	0.02	0.03
Block-matching	CR	MRI	2.83	0.03	0.15
Block-matching	NCC	Pseudo US	2.52	0.81	
Intra-rater error			2.14		0.81

## Footnotes

1 http://www.intergrowth21.org.uk.

2 http://www.interbio21.org.uk.

3 http://www.doc.ic.ac.uk/∼dr/software.

4 We thank the Department of Neonatal Imaging, Imperial College London for providing these datasets.

5 http://www.brain-development.org.

6 We thank the Intergrowth-21st Consortium for providing the US images.

7 http://www.doc.ic.ac.uk/∼dr/software.
